# Effects of dietary crude protein levels on growth performance, rumen characteristics, blood metabolites, and methane emissions in finishing Hanwoo steers

**DOI:** 10.5713/ab.25.0063

**Published:** 2025-04-28

**Authors:** Joonpyo Oh, Hyunjin Cho, Namkyu Kang, Mingyung Lee, Md Raihanul Hoque, Yoo Yong Kim, Seongwon Seo

**Affiliations:** 1 Cargill Animal Nutrition Korea, Seongnam, Korea; 2 Division of Animal and Dairy Sciences, Chungnam National University, Daejeon, Korea; 3 Department of Agricultural Biotechnology and Research Institute of Agriculture and Life Sciences, Seoul National University, Seoul, Korea

**Keywords:** Crude Protein, Finishing Steers, Hanwoo, Weight Gain

## Abstract

**Objective:**

The objective of this study was to investigate the effects of increasing dietary crude protein (CP) concentration on growth performance, rumen characteristics, blood metabolites, and methane (CH_4_) emissions in finishing Hanwoo steers.

**Methods:**

Twenty-four 26-month-old Hanwoo steers (717±50.1 kg) were utilized in a 12-week study based on a completely randomized block design. The animals were randomly assigned to one of four dietary treatments by feeding concentrate mixes with different CP levels (15.0%, 16.2%, 17.5%, and 18.5% on a dry matter [DM] basis). Forage was fed ad libitum, while the concentrate mix was provided in a fixed amount.

**Results:**

Forage and total dry matter intake (DMI) linearly increased (p≤0.029) with increasing CP levels while concentrate intake was not affected by treatments. Initial and final body weight and average daily gain were not different among treatments. Feed efficiency showed a trend of linear decrease (p = 0.092) with increasing CP levels. Rumen parameters, including ruminal pH, total volatile fatty acid, molar proportions of acetate, propionate, isobutyrate, butyrate, isobutyrate, valerate, and acetate to propionate ratio, were not affected by treatments. Treatments also did not affect blood concentrations of total protein, urea, glucose, non-esterified fatty acid, albumin, creatinine, triglyceride, glutamic oxaloacetic transaminase, glutamic pyruvic transaminase, calcium, and phosphorus. Concentrations of CH_4_ from respiration or eructation were not affected by dietary CP. CH_4_ ppm per DMI and neutral detergent fiber (NDF) intake was not also different among treatment in respiration or eructation. However, CH_4_ ppm per forage NDF intake linearly decreased (p≤0.005) with increasing CP from both respiration and eructation.

**Conclusion:**

These findings provide no clear evidence that increasing the CP content of the concentrate mix from 15.0% to 18.5% DM improves growth performance and body metabolism in finishing Hanwoo steers. Nevertheless, they suggest that a higher CP level may negatively affect feed efficiency.

## INTRODUCTION

Crude protein (CP) is a fundamental component in beef cattle nutrition, essential for various physiological functions including growth, reproduction, lactation, and health [1,2]. It comprises both true protein and non-protein nitrogen sources such as urea, which rumen microbes utilize to synthesize microbial protein-a major contributor to the metabolizable protein available to cattle [3].

Studies have been conducted to investigate the effects of CP levels on growth performance in beef cattle. Xia et al [4] reported that average daily gain (ADG) and feed efficiency were linearly increased with increasing dietary CP from 10.2% to 14.2% dry matter (DM) in growing beef cattle. Positive effects on weight gain with higher CP diets were also observed in studies with finishing beef cattle [5,6]. However, Gleghorn et al [7] and Bailey et al [8] reported quadratic increases in ADG of finishing steers fed increasing CP diets from 11.5% to 14.5% and 11.0% to 14.0% DM, respectively. Studies in Hanwoo cattle, a Korean native breed, are limited and results have been inconsistent. Jeong et al [9] reported that a higher CP diet (14.0% DM) increased ADG compared to a lower CP diet (12.0% DM) in finishing Hanwoo steers. In a recent study, however, no effect was found in ADG between 12.0% and 15.0% CP diets in finishing Hanwoo steers [10].

The Ministry of Agriculture, Food and Rural Affairs in Korea recently set a maximum limit in CP level of commercial concentrates as 15% on a as-fed basis—approximately 17% DM— for finishing Hanwoo beef cattle over 500 kg body weight (BW) in the control of livestock and fish feed act [11]. It was intended by the action to prevent excessive CP in beef cattle feeds which can increase nitrogen excretion into the environment and contribute to ammonia (NH_3_-N) emissions [2]. However, it has not been studied in finishing Hanwoo steers how dietary CP level in commercial concentrates over 17% DM affects growth performance and body metabolism. Thus, the objective of this study is to investigate the effects of increasing dietary CP levels from 15.0% to 18.5% DM—over 17% DM—in the concentrate mix on feed intake, weight gain, rumen fermentation, blood metabolites, and enteric methane (CH_4_) emissions in finishing Hanwoo steers.

## MATERIALS AND METHODS

This study was conducted at the Center for Animal Science Research, Chungnam National University, Korea. The use of animals and the protocols for this experiment were reviewed and pre-approved by the Chungnam National University Animal Research Ethics Committee (202203A-CNU-059).

### Animals, housing, and diets

Twenty four 26-month-old Hanwoo finishing steers (717± 50.1 kg), blocked by initial BW and estimated breeding values for carcass BW were randomly assigned to one of four dietary treatments based on completly radomized design [12]. Six steers of similar BW were grouped within a block and housed together in a pen (10 m×10 m) equipped with four forage intake monitoring systems (FIMS) and an automatic concentrate feeding station (ACFS). FIMS and ACFS allowed us to measure individual feed intake (kg/day) automatically by identifying each animal using a radio-frequency identification tag attached to them (Dawoon, Incheon, Korea). The experiment was conducted for three months, with a 7-day adaptation period beforehand.

Two types of concentrate mix were prepared for the study: a low CP concentrate mix containing CP of 15.0% based on DM and a high CP concentrate mix containing CP of 18.5% based on DM. These were combined in varying proportions to create four dietary treatments with incremental CP levels: 1) low CP (100% low CP concentrate mix), 2) medium low CP (65% low CP contrate mix and 35% high CP concentrate mix), 3) medium-high CP (30% low CP concentrate mix and 70% high CP concentrate mix), and 4) high CP (100% high CP concentrate mix). The diets were made to meet the animal nutrient requirement for ADG of 0.9 kg/day, based on the Korean feeding standards for Hanwoo growing steers [13]. Feedings were scheduled at 08:00 and 18:00 h. Tall fescue, provided as forage, and drinking water were available *ad libitum* throughout the experiment. The concentrate mix was provided through the ACFS. Each day was divided into six-time intervals of 4 h each, and the steers were able to consume up to one-sixth of the daily allowable concentrate mix (8.5 kg/day). If the steers did not consume the amount of concentrate mix allowed during each interval, they could consume the rest during the following interval. Detailed formulations and chemical compositions of the experimental diets are presented in Tables 1, 2.

### Measurement and sample collection

Forage and concenctrate mix daily intake were recorded individually through the FIMS and ACFS (Dawoon). The total dry matter intake (DMI) was calculated as the sum of the separately recorded forage and concentrate intake. Every four weeks, each steer’s recorded daily feed intakes were processed. Intakes with more than or less than three times the SD from the mean intake were treated as outliers and removed. The intakes of the day when management could affect feed intakes, such as bedding replacement, BW measurement, and sampling periods, took place were also removed. The BW of steers was mesured every four weeks before morning feeding and the feed samples were sampled once every four weeks.

Rumen fluid was collected three times (−1, +3, and +6 h after morning feeding) on three consecutive days after 5 weeks and 11 weeks using an oral stomach tube as described [14]. Briefly, the initially collected rumen fluid was discarded (approximately 300 mL), and 400 mL of rumen fluid was collected in a glass flask. After collection, the pH of the rumen fluid was measured immediately, and 10 mL each was subsampled for volatile fatty acid (VFA) analysis and stored at −20°C until analysis.

About 10 mL of blood was collected from the jugular vein of the all steers before morning feeding after 12 weeks. The collected blood was into a serum seperator tube (BD Vacutainer; BD and Co., Franklin Lakes, NJ, USA). Serum was obtained by centrifugation at 1,300×g for 15 min at 4°C and frozen at −80°C until analysis.

CH_4_ emissions of all steers were measured four times (−2, −1, +1, and +2 h after morning feeding) for five consecutive days using a laser methane detector (LMD) after 10 weeks as described Kang et al [15] and duplicated for an additional five consecutive days. Briefly, with the LMD installed stably on a tripod, the visible laser was aimed at the steer’s nose from a distance of 1 m, and CH_4_ emissions were measured every 0.5 s for 6 min.

### Sample analyses

Two steers were excluded from all analyzes due to health concerns. The feed samples were dried at 60°C for 96 h and ground through a cyclone mill (Foss, Hillerød, Denmark) fitted with a 1 mm screen. The nutrient composition of the feed samples was analyzed at Cumberland Valley Analytical Services (Hagerstown, MD, USA). The details of the methods used to analyze the feed samples’ nutrient content were the same as described in Jeon et al [16].

In order to determine the VFA concentration of rumen fluid, rumen fluid supernatant (1 mL) was mixed with 0.2 mL of metaphosphoric acid (250 g/L) and kept at 4°C for 30 min. Following centrifugation of the mixture at 21,000×g for 10 min at 20°C, the supernatant was injected into a gas chromatograph (HP 6890, Hewlett-Packard, Palo Alto, CA, USA) equipped with a flame ionization detector and capillary column (Nukol Fused silica capillary column 30 m×0.25 mm× 0.2 μm, Supelco, Bellefonte, PA, USA). The temperature of the oven, injector, and detector was 90°C to 180°C, 185°C, and 210°C, respectively. Nitrogen was used as the carrier gas at a flow rate of 40 mL/min. The serum was analyzed for total protein (TP), aspartate transaminase, alanine transaminase, glucose, total cholesterol, triglycerides, non-esterified fatty acid (NEFA), blood urea nitrogen (BUN), creatinine, calcium (Ca), inorganic phosphate, and albumin using kits purchased from Wako Pure Chemical Industries (Osaka, Japan) and a clinical auto analyzer (Toshiba Accute Biochemical Analyzer-TBA-40FR, Toshiba Medical Instruments, Tokyo, Japan).

CH_4_ emission data were separated into respiration and eructation by detecting CH_4_ concentration peaks using the automatic multi-scale peak detection pakage in R software [17] and fitting a double normal distribution using the mixdist pakage in R software. The mean of a normal distribution represents the mean CH_4_ concentration per day as the mean of four time-of-day values, assumed representative CH_4_ concentrations of exhaled gas from the pathway during that time period [13].

### Statistical analysis

All data were analyzed using PROC MIXED procedure of SAS (SAS Institute, Cary, NC, USA). The blocks (i.e., initial BW and breeding value for carcass weight) were treated as random effects. Individual means were also compared by a Tukey’s test. To test the effects of treatments on rumen parameters, the data were analyzed as repeated measures to account for the correlation between repeated measurements of each animal. For this analysis, no structure was assumed for the variance-covariance matrix. Significance was declared at p<0.05, and a trend was discussed at 0.05≤ p<0.1.

## RESULTS AND DISCUSSION

Feed intake and animal performance data are shown in Table 3. Forage and total DMI linearly increased (p≤0.029) with increasing CP levels while concentrate intake was not affected by treatments. Initial and final BW were not different among treatments. ADG was numerically decreased as dietary CP increased, but it was not statistically significant (p = 0.154) due to a large variation. There was a trend of linear increase (p = 0.092) in feed conversion ratio (FCR) with treatments. The effects of dietary CP level on feed intake are inconsistent among studies in beef cattle. Gleghorn et al [7] and Boonsaen et al [18] reported that increasing CP concentration from 11.5% to 14.5% DM did not affect DMI in different breeds of finishing steers. In studies with Hanwoo steers, DMI was not affected by various CP levels from 12% to 17.3% DM in finishing phase [9,19]. However, Bailey et al [8] obvered that DMI was quadratically increased with three differenct CP levels (11%, 12.5%, and 14.0% DM) in finishing steers. This quadratic increase resulted from the intake reduction in the diet of 11% concentrate CP, which might cause limitation in rumen degradable protein (RDP) supply [8]. This might be not the case in the current study because all the treatments were formulated to meet the nutrient requirement. Increase in DMI in the current study was mainly due to higher forage intake which was fed ad libitum. It is unclear how higher CP diet increased forage intake. It is possibly thought that numerically higher intake of concentrate in higher CP diets could lead to a lower ruminal pH after consuming concentrate mix, so that it might lead to higher voluntary intake of forage in those cattle [20]. However, ruminal pH in the current study was not different among treatments, which is discussed later in this paper. The tendency of linear increase in FCR was mainly attributed to the increase in DMI in higher CP diets because weight gain was not affected by the treatment in the current study. The effects of different CP levels on weight gain or ADG in beef cattle have been varied among studies. Archibeque et al [5] reported increases in ADG for medium (11.8% DM) and high (14.9% DM) CP diets compared with a low (9.1% DM) CP diet in a 212 d feeding trial with finishing steers. Similar results were observed in a study with finishing bulls that a 15.0% CP diet increased ADG compared with 13.5% [6]. The authors concluded that higher ADG in the 15.0% CP diet resulted from higher DMI than the lower CP diet. Bailey et al [8] reported that increasing CP levels (11.0%, 12.5%, and 14.0% DM) quadratically increased ADG in a study with finishing beef steers and heifers. Similarly with the study by Cortese et al [6], the improvement of ADG was attributed to a quadratic increase of DMI in this study. In the current experiment with Hanwoo cattle, however, no positive effect was observed in ADG with increasing CP concentration. This is in agreement with a study by Jeon et al [19] who reported that a higher CP diet (17.3% DM) did not affect ADG compared to the control (15.6% DM) in Hanwoo beef cattle. Lee et al [10] also demonstrated in their study with finishing Hanwoo steers that ADG was not different between a 15.0% and 12.0% CP diets. It should be noted that the feeding period for Hanwoo beef production in Korea is relatively longer than that of other countries in North and South America and Europe. The ‘finishing’ phase of Hanwoo beef steers is typically considered from 23 to 30 (being slaughtered) months of age. It is plausible that no effect of increasing CP on ADG in the current experiment was attributed to older ages of Hanwoo steers than the animals in the previously mentioned studies [6–8].

Rumen parameters in this study, including ruminal pH, total VFA, molar proportions of acetate, propionate, isobutyrate, butyrate, isobutyrate, valerate, and acetate to propionate ratio, were not affected by treatments (Table 4). Ruminal pH in the current experiment was comparable to that of other studies in beef cattle [4,21,22]. Similar to the current study, Chen et al [21] and Oh et al [22] found no effect of increasing CP on ruminal pH in beef cattle. However, Xia et al [4] observed a linear increase in ruminal pH with increasing CP diets (from 10.21% to 14.24% DM) in Holstein bulls. The authors pointed out that the linear increase of ruminal pH was due to increased ruminal NH_3_-N concentration by treatments. A positive correlation was found in that study between ruminal pH and NH_3_-N concentration. Oppositely, ruminal pH was linearly decreased by increasing dietary CP levels from 9.2 to 21.9 DM although ruminal NH_3_-N was increased in another study with buffaloes [23]. Ruminal VFA production also varied among studies investigating the effects of different CP levels. A meta-analysis study using data from *in vitro* continuous culture systems reported that dietary CP quadratically increased total VFA and propionate concentrations [24], in which total VFA was the highest at 18% CP. However, Van Dung et al [25] found no effect of different CP treatemnts (from 10% to 19% DM) on total VFA, acetate, propionate, or butyrate concentrations in an *in vitro* batch culture system. *In vivo* studies with beef cattle by Oh et al [26] and Chen et al [21] showed that total VFA, acetate, propionate, and butyrate concentrations were not changed by increasing CP, which is in agreement with the current study. However, Xia et al [4] observed increases in total VFA, acetate, and propionate concentrations with increasing CP in Holstein bulls, and Oh et al [22] reported that increasing CP decreased ruminal propionate concentration without effect on total VFA in Hanwoo steers. The inconsistent results in ruminal pH and VFA concentration might be due to discrepancies in beef breeds, growth stages, and feed ingredients across studies.

Blood concentrations of TP, urea, glucose, NEFA, albumin, creatinine, triglyceride, glutamic oxaloacetic transaminase, glutamic pyruvic transaminase, Ca, and phosphorus were not affected by treatments (Table 5). It was also found in other beef cattle studies that TP, albumin, and creatinine concentrations in blood were not affected by dietary CP levels [10,26]. Unlike the current study, BUN was linearly increased by increasing CP levels in other studies with buffaloes or Holestein bulls [4,23]. Chanthakhoun et al [23] indicated in their study that excessive N availability in the rumen of cattle fed higher CP diets increased NH_3_-N absorption into blood, resulting in higher conversion to urea in the liver. This is in line with a study by Javaid et al [27] who observed a linear increase in BUN with increasing RDP proportion from 50% to 100% in a study with buffalo bulls. Lee et al [10] also reported that a higher RDP diet increased BUN concentration compared to a lower RDP diet in Hanwoo steers on the fattening phase. Interestingly, BUN was not affected by RDP levels in the finishing phase in the same study, which is similar to the results in the current study with finishing Hanwoo steers. Readily degradable protein fraction (A fraction+B1 fraction) in the current study was numerically increased as dietary CP increased (Table 2), but BUN was not affected by treatments.

Concentrations of CH_4_ from respiration or eructation were not affected by dietary CP alteration (Table 6). CH_4_ ppm per DMI and neutral detergent fiber (NDF) intake was not also different among treatments in respiration or eructation. However, CH_4_ ppm per forage NDF intake linearly decreased (p≤0.005) with increasing CP from both respiration and eructation. This is apparently because of increased forage intake in higher CP diets (Table 3). Studies investigating the effects of dietary CP on enteric CH_4_ emission are limited in beef cattle. Oh et al [22] reported that CH_4_ concentration (ppm) from eructation tended to decrease with increasing dietary CP in Hanwoo steers on the fattening stage. CH_4_ concentration per DMI, forage NDF intake, NDF intake, and ADG were also linearly decreased. However, the authors indicated in the study that the CH_4_ mitigation effects of higher CP diets were attributed to feed ingredients rather than CP concentration itself. Indeed, studies with dairy cattle were inconsistent in the effects of varying CP concentrations on enteric CH_4_ emissions [28–30]. Further studies are needed to investigate the interaction between dietary CP levels and feed ingredients on enteric CH_4_ emission in cattle.

## CONCLUSION

In conclusion, increasing dietary CP content from 15.0% to 18.5% DM in the concentrate mix did not enhance growth performance in finishing Hanwoo steers although forage intake was linearly increased by the treatment. Ruminal pH, VFA production, blood metabolites, and enteric CH_4_ emissions were not affected by the different CP levels. Contrary to expectations, providing finishing Hanwoo steers with a high-CP concentrate mix exceeding 17% DM is unlikely to improve productivity and may instead reduce feed efficiency.

## Figures and Tables

**Table 1:** Diet formulation of the experimental concentrate mix

Items	Treatment^1)^			

	LCP	MLCP	MHCP	HCP
Ingredients (g/kg DM)				
Corn (flaked)	300	300	300	300
Corn (ground)	282	232	181	138
Wheat (ground)	50	68	85	100
Palm oil	2	2	2	2
Rice bran	33	39	45	50
Corn gluten feed	5	6	8	9
DDGS	33	75	117	154
Soybean meal	0	9	19	27
Palm kernel meal	186	156	126	100
Molasses	58	55	51	48
CMS	30	30	30	30
Urea	0	2	3	5
Sodium bicarbonate	4	5	5	6
Limestone	14	19	24	29
Salt	2	2	2	2
Vitamin and mineral mix^2)^	1	1	1	1

1) LCP, low crude protein (15.0%); MLCP, middle-low crude protein (16.2%); MHCP, middle-high crude protein (17.5%); HCP, high crude protein (18.5%).

2) 33,330,000 IU/kg vitamin A, 40,000,000 IU/kg vitamin D, 20.86 IU/kg vitamin E, 20 mg/kg Cu, 90 mg/kg Mn, 100 mg/kg Zn, 250 mg/kg Fe, 0.4 mg/kg I, and 0.4 mg/kg Se.

DDGS, dried distiller’s grains with soluble; CMS, condensed molasses fermentation soluble.

**Table 2:** Analyzed chemical composition (g/kg DM or as stated) of the experimental diets

Items	Treatment^1)^				Tall fescue

	LCP	MLCP	MHCP	HCP	
DM (g/kg as fed)	882	885	888	891	895
OM	926	925	924	923	930
CP	150	162	175	185	63
SOLP	60	68	76	82	23
NDICP	25	24	24	23	15
ADICP	9	9	10	10	7
aNDF	262	256	249	244	742
ADF	106	106	107	107	439
ADL	38	37	37	36	64
Ether extract	47	48	48	49	11
Ash	74	75	76	77	70
Ca	14	15	16	17	3
P	5	5	6	6	1
K	8	8	8	8	23
Na	4	4	3	3	1
Cl	4	4	3	3	6
S	3	3	4	4	1
Mg	3	3	3	3	2
TDN	740	743	746	748	518
NEm (MJ/kg DM)	8.0	8.0	8.1	8.1	4.2
NEg (MJ/kg DM)	5.3	5.3	5.4	5.4	1.9
Total carbohydrates	730	715	700	688	856
NFC	493	485	476	469	126
Carbohydrate fraction (g/kg carbohydrate)					
CA	71	77	84	89	77
CB1	508	486	465	447	4
CB2	95	113	130	145	67
CB3	199	198	196	195	672
CC	126	126	125	125	178
Protein fraction (g/kg CP)					
PA+B1	397	413	429	443	365
PB2	438	435	432	430	405
PB3	103	93	83	75	116
PC	62	58	54	51	114

1) LCP, low crude protein (15.0%); MLCP, middle-low crude protein (16.2%); MHCP, middle-high crude protein (17.5%); HCP, high crude protein (18.5%).

DM, dry matter; OM, organic matter; CP, crude protein; SOLP, soluble CP; NDICP, neutral detergent insoluble CP; ADICP, acid detergent insoluble CP; aNDF, neutral detergent fiber analyzed using a heat stable amylase and expressed inclusive of residual ash; ADF, acid detergent fiber; ADL, acid detergent lignin; TDN, total digestible nutrients; NEm, net energy for maintenance; NEg, net energy for growth; NFC, non-fiber carbohydrate; CA, carbohydrate A fraction; ethanol soluble carbohydrates; CB1, carbohydrate B1 fraction; starch; CB2, carbohydrate B2 fraction; soluble fiber; CB3, carbohydrate B3 fraction; available insoluble fiber; CC, carbohydrate C fraction; unavailable carbohydrate; PA+B1, protein A and B1 fractions; soluble CP; PB2, protein B2 fraction; intermediate degradable CP; PB3, protein B3 fraction; slowly degradable fiber-bound CP; PC, protein C fraction; unavailable CP.

**Table 3:** Effects of dietary crude protein levels of concentrate mix on growth performance in finishing Hanwoo steers

Items	Treatment^1)^				SEM	p-value		
	
	LCP	MLCP	MHCP	HCP		Mean	Linear	Quadratic
DMI (kg/day)								
Concentrate	6.97	7.04	6.64	7.15	0.262	0.567	0.898	0.420
Forage	1.40	2.15	2.08	2.49	0.266	0.056	0.014	0.530
Total	8.37	9.20	8.72	9.64	0.318	0.053	0.029	0.893
CPI (kg/day)	1.34^c^	1.62^b^	1.73^ab^	1.95^a^	0.061	<0.001	<0.001	0.588
Initial BW (kg)	716	717	718	719	22.0	1.000	0.918	0.993
Final BW (kg)	782	766	768	752	21.9	0.824	0.939	0.999
ADG (g/day)	592	441	449	304	129.1	0.493	0.154	0.985
FCR (DMI [g]/ADG [g])	15.1	23.4	10.0	54.6	12.72	0.092	0.080	0.169

1) LCP, low crude protein (15.0%); MLCP, middle-low crude protein (16.2%); MHCP, middle-high crude protein (17.5%); HCP, high crude protein (18.5%).

a–c Means that do not have common superscripts significantly differ within the treatments (p<0.05).

SEM, standard error of the mean; DMI, dry matter intake; CPI, crude protein intake; BW, body weight; ADG, average daily gain; FCR, feed conversion ratio.

**Table 4:** Effects of dietary crude protein levels of concentrate mix on rumen characteristics in finishing Hanwoo steers

Items	Treatment^1)^				SEM	p-value		
	
	LCP	MLCP	MHCP	HCP		Mean	Linear	Quadratic
pH	6.74	6.67	6.70	6.69	0.080	0.942	0.770	0.725
Total VFA (mM)	58.09	60.81	58.85	58.84	3.212	0.940	0.956	0.676
Molar proportions (mmol/mol)								
Acetate	622	610	616	613	14.5	0.935	0.735	0.756
Propionate	185	196	202	200	7.4	0.399	0.141	0.402
Isobutyrate	25	24	24	23	1.4	0.928	0.574	0.788
Butyrate	117	121	111	117	6.1	0.694	0.727	0.842
Isovalerate	28	29	27	26	2.6	0.805	0.416	0.733
Valerate	23	21	21	21	1.1	0.554	0.269	0.376
Acetate/Propionate	3.4	3.2	3.1	3.1	0.18	0.604	0.232	0.571

1) LCP, low crude protein (15.0%); MLCP, middle-low crude protein (16.2%); MHCP, middle-high crude protein (17.5%); HCP, high crude protein (18.5%).

SEM, standard error of the mean; VFA, volatile fatty acid.

**Table 5:** Effects of dietary crude protein levels of concentrate mix on blood metabolites in finishing Hanwoo steers

	Treatment^1)^				SEM	p-value		
	
Items	LCP	MLCP	MHCP	HCP		Mean	Linear	Quadratic
Total protein (g/dL)	7.6	7.5	7.6	7.3	0.15	0.607	0.287	0.719
Urea (mg/dL)	12.6	12.2	13.1	12.5	0.92	0.932	0.931	0.932
Glucose (mg/dL)	65.9	68.5	68.8	66.1	1.86	0.581	0.929	0.173
NEFA (mEq/L)	0.35	0.36	0.38	0.32	0.037	0.746	0.657	0.378
Albumin (mg/dL)	3.5	3.7	3.6	3.6	0.06	0.203	0.470	0.189
Creatinine (mg/dL)	1.2	1.2	1.4	1.3	0.06	0.124	0.149	0.839
Triglyceride (mg/dL)	16.3	13.9	14.8	16.6	2.32	0.823	0.880	0.371
GOT (U/L)	68.8	58.6	58.9	61.8	3.52	0.684	0.723	0.260
GPT (U/L)	17.2	18.7	18.3	18.5	1.23	0.823	0.521	0.616
Cholesterol (mg/dL)	177.8	179.3	189.0	172.4	10.43	0.727	0.895	0.396
Calcium (mg/dL)	8.6	8.4	8.6	8.4	0.10	0.359	0.380	0.863
Phosphorus (mg/dL)	6.0	6.4	5.7	6.0	0.21	0.242	0.477	0.859

1) LCP, low crude protein (15.0%); MLCP, middle-low crude protein (16.2%); MHCP, middle-high crude protein (17.5%); HCP, high crude protein (18.5%).

SEM, standard error of the mean; NEFA, non-esterified fatty acid; GOT, glutamic oxaloacetic transaminase; GPT, glutamic pyruvic transaminase.

**Table 6:** Effects of dietary crude protein levels of concentrate mix on methane emissions in finishing Hanwoo steers

Items	Treatment^1)^				SEM	p-value		
	
	LCP	MLCP	MHCP	HCP		Mean	Linear	Quadratic
CH_4_ from respiration								
ppm	20.4	19.6	20.3	20.2	2.03	0.992	0.973	0.875
ppm/kg of DMI	2.4	2.1	2.4	2.0	0.26	0.613	0.494	0.784
ppm/kg of FNDFI	26.8^a^	13.9^ab^	16.3^ab^	10.4^b^	3.30	0.013	0.005	0.301
ppm/kg of NDFI	7.1	5.8	6.8	5.3	0.76	0.327	0.195	0.915
CH_4_ from eructation								
ppm	91.9	83.4	84.1	84.1	11.06	0.939	0.651	0.702
ppm/kg of DMI	10.7	8.9	9.8	8.2	1.28	0.561	0.270	0.930
ppm/kg of FNDFI	118.3^a^	59.5^b^	65.8^ab^	42.3^b^	14.73	0.010	0.003	0.244
ppm/kg of NDFI	32.5	25.0	27.6	21.9	3.81	0.281	0.105	0.816

1) LCP, low crude protein (15.0%); MLCP, middle-low crude protein (16.2%); mHCP, middle-high crude protein (17.5%); HCP, high crude protein (18.5%).

a,b Means that do not have common superscripts significantly differ within the treatments (p<0.05).

SEM, standard error of the mean; DMI, dry matter intake; FNDFI, forage neutral detergent fiber intake; NDFI, neutral detergent fiber intake.
